# Electrohydrodynamic Processing of PVP-Doped Kraft Lignin Micro- and Nano-Structures and Application of Electrospun Nanofiber Templates to Produce Oleogels

**DOI:** 10.3390/polym13132206

**Published:** 2021-07-03

**Authors:** José F. Rubio-Valle, M. C. Sánchez, Concepción Valencia, José E. Martín-Alfonso, José M. Franco

**Affiliations:** Pro2TecS—Chemical Process and Product Technology Research Centre, Departamento Ingeniería Química, ETSI, Campus de “El Carmen”, Universidad de Huelva, 21071 Huelva, Spain; josefernando.rubio@diq.uhu.es (J.F.R.-V.); mcarmen@uhu.es (M.C.S.); jose.martin@diq.uhu.es (J.E.M.-A.); franco@uhu.es (J.M.F.)

**Keywords:** eucalyptus kraft lignin, PVP, electrospinning, nanofibers, oleogel, rheology

## Abstract

The present work focuses on the development of lignin micro- and nano-structures obtained by means of electrohydrodynamic techniques aimed to be potentially applicable as thickening or structuring agents in vegetable oils. The micro- and nano-structures used were mainly composed of eucalyptus kraft lignin (EKL), which were doped to some extent with polyvinylpyrrolidone (PVP). EKL/PVP solutions were prepared at different concentrations (10–40 wt.%) and EKL:PVP ratios (95:5–100:0) in *N, N*-dimethylformamide (DMF) and further physico-chemically and rheologically characterized. Electrosprayed micro-sized particles were obtained from solutions with low EKL/PVP concentrations (10 and 20 wt.%) and/or high EKL:PVP ratios, whereas beaded nanofiber mats were produced by increasing the solution concentration and/or decreasing EKL:PVP ratio, as a consequence of improved extensional viscoelastic properties. EKL/PVP electrospun nanofibers were able to form oleogels by simply dispersing them into castor oil at nanofiber concentrations higher than 15 wt.%. The rheological properties of these oleogels were assessed by means of small-amplitude oscillatory shear (SAOS) and viscous flow tests. The values of SAOS functions and viscosity depended on both the nanofiber concentration and the morphology of nanofiber templates and resemble those exhibited by commercial lubricating greases made from traditional metallic soaps and mineral oils.

## 1. Introduction

In the last decades, there has been a drive to develop sustainable and environmentally friendly products to reduce dependence on fossil fuels. The urgency to drastically reduce petroleum-derived plastics from the ecosystem has triggered extensive research into their replacement with renewable materials and biopolymers [1]. Several industries, such as those involved in the production of lubricants [2], face serious environmental problems [3] and demand robust and efficient eco-friendly oleogel-like products with suitable functional properties, which can overcome these problems. Thus, the amount of scientific and technical research related to the development of bio-lubricants from natural resources has increased exponentially in the last two decades [4,5,6]. In this sense, the combined use of natural polymers and vegetable oils is proposed to develop oleogels or stable dispersions [3,7,8,9]. This stabilization can be achieved physically, by reducing the polymer polarity, making them more similar to the oil medium—for example by inserting methyl, ethyl, or acyl groups into their structure—or chemically, by functionalization with reactive groups able to produce covalent interactions with the oil, thus promoting a certain degree of cross-linking between medium and thickener. The latter is the most efficient way to develop formulations that are technically competitive with conventional lubricating greases. However, it requires relatively complex chemical modifications that make the process of obtaining formulations not completely environmentally friendly, although the final product is [10,11,12,13,14]. On the other hand, based on the concept of biorefinery as a global industrial process capable of producing intermediate and end-use products from biomass in a versatile way [15], the interest in the use of lignocellulose as a raw material in the chemical and energy industries has been steadily increasing in recent years [16]. Unlike other biomasses used as raw materials to obtain natural compounds such as sugars or starch, lignocellulosic biomass is low-cost, abundant, widely distributed, and not intended for food consumption. Many different forest species such as those of the *Myrtaceae* and *Oleaceae* families are potentially suitable for lignocellulosic biomass production. Cellulose is the most appreciated lignocellulose component, nowadays employed in a wide variety of high-performance and/or high-technology materials [17,18,19,20]. However, for other lignocellulose components, such as hemicellulose or lignin, not as many means of valorization have been found, and they are even considered by-products or waste products. This is the case for lignin, which contains a high carbon content (>60 wt.%) arising from the phenylpropane (C9) structure, thus showing a much higher yield of carbon relative to other abundant natural biopolymers such as cellulose (~45% carbon content). Aiming to find different valorization pathways for lignin, some researchers have been studying the conversion of lignin into carbon fibers [21,22], and some technical lignins have been successfully processed into microscale fibers through melt spinning or gel spinning techniques [23,24,25]. In this sense, electrospinning is a well-established technique for the fabrication of micro- and nano-sized polymer fibers. In contrast to other fiber-spinning techniques such as melt spinning or dry spinning, which rely on mechanical forces to produce fibers by drawing the polymer through a spinneret, this technique relies on electrostatic forces [26,27]. Electrospun lignin fibers can achieve nanoscale diameters and be deposited as a nonwoven or aligned fiber [28]. As is well-known, in this process, a strong electrical field is applied during spinning causing a jet of the polymer solution to be stretched. The jet elongates so much that it can form nanoscale diameter fibers with chains possessing molecular alignment [29].

Several studies reported the electrohydrodynamic production of lignin nanofibers using a dopant polymer, such as polyethylene oxide (PEO), polyvinyl alcohol (PVA), or polyacrylonitrile (PAN), among others, to improve electrospinnanbility, thus obtaining homogeneous and bead-free nanofibers. Dallmeyer et al. [24] produced technical lignin nanofibers via electrospinning using different types of technical lignins with the addition of PEO. The analysis of the different morphologies obtained allowed them to conclude that none of the lignins could form nanofibers without adding PEO, but instead, only the formation of microbeads occurred. PAN and PVA are also other doping polymers that have been used to obtain fiber architectures from lignins [30,31]. In general, pure lignin solution (without additives or doping polymers) cannot form continuous and homogeneous fiber mats, but rather, typically a beaded structure [32,33]. In most studies, by adding small amounts of a dopant polymer, lignin can be electrospun into nanofibers with uniform morphology without defects [24,34]. These fibers have significant advantages for several applications such as being extremely lightweight, and their low-cost production, large aspect ratio with reduced defects (small and uniform diameters), high molecular orientation, and flexibility in surface functionality [27,35]. Others researchers have been succeeded in producing electrospun lignin fibers, but each study focused on a single type of lignin, hence it is difficult to translate the findings related to one type of lignin to another [36,37,38].

On the other hand, structuring or gelling oils physically with natural polymers, i.e., without any chemical modification that modifies the solvent-biopolymer hydrophobic interactions [9], cannot be achieved except for a few biopolymers such as ethylcellulose [39] However, considering the dried foam-template oleogelation mechanism [40,41], where the oil is absorbed in the generated voids of large available surface networks, herein, it is hypothesized that the high porosity, small size and high surface/volume ratio of nanostructures obtained by electrospinning may induce the formation of three-dimensional networks, which may promote the required physical interactions between the oil and the nanostructures to physically stabilize the oleogel. Previous studies have shown limited physical stability of biopolymer-thickened oleogels against phase separation, presenting syneresis problems, namely the so-called “oil bleeding”, after a relatively short period of ageing [8,42]. Thus, the conventional processing route, i.e., thermo-mechanical treatments to properly disperse the biopolymer into the oil, inevitably provides oleogels with a series of fundamental problems or limitations, which may be overcome by producing biopolymer nanostructures. In this work, novel and green oleogel formulations were prepared using electrospun lignin nanostructures and castor oil. For this purpose, micro and nano-structures were produced by electrohydrodynamic processing from solutions of eucalyptus kraft lignin (EKL) in *N, N*-dimethylformamide (DMF) doped with polyvinylpyrrolidone (PVP). The morphology of electrospun EKL/PVP nanostructures was related to the rheological properties of resulting oleogels. Finally, these oleogels are proposed as potential substitutes for traditional lubricating greases.

## 2. Materials and Methods

### 2.1. Materials

Eucalyptus kraft lignin (EKL) and polyvinylpyrrolidone (PVP) were used as raw materials. EKL comes from *Eucalyptus globulus* wood cooking ENCE S.A. (Madrid, Spain). Extensive information about the kraft lignin isolation processes and structural characterization can be seen elsewhere [43]. EKL was kindly supplied by INIA-CIFOR. PVP (Mw: 360,000 g/mol) and DMF (purity ≥ 99.8%) were provided by Merck Sigma Aldrich S.A. (Taufkirchen, Germany). Castor oil from Guinama (Valencia, Spain) was used as an oil medium to prepare oleogels. Fatty acid composition and the main physical properties of this vegetal oil can be found elsewhere [44].

### 2.2. Preparation of EKL/PVP Solutions

EKL and EKL/PVP solutions in DMF were manufactured at different mass fractions (10, 20, 30 and 40 wt.%) and the different EKL/PVP ratios are indicated in Table 1. All the solutions were prepared using a magnetic stirrer (500 rpm), at room temperature, for 24 h.

### 2.3. Electrospinning

Electrospinning of EKL/PVP solutions was performed in a DOXA Microfluidics (Málaga, Spain) chamber. This electrospinning device consists of a flow pump, an aluminum plate collector, a Spellman high voltage source, and a camera monitor to visualize the correct formation of the Taylor cone. For the electrospinning process, 15 mL of the spinning solution was placed in a plastic syringe fitted with an 18-G needle. The syringe was attached to the holder using a vertical configuration and 15 kV was supplied by the high voltage power source. A distance of 17 cm was established between the aluminum collector plate (cathode) and the needle tip (anode). A positively charged jet from the tip of the syringe is projected onto the negatively charged collector. The nanostructure was formed on the collector plate and then carefully removed. All experiments were performed at room temperature (22 ± 1 °C) and almost constant relative humidity (45 ± 1%).

### 2.4. Characterization of EKL/PVP Solutions

Steady-state shear viscosity measurements of EKL/PVP solutions were carried out using an ARES (Rheometric Scientific, Leatherhead, UK) controlled-strain rheometer, in a shear rate range of 1–300 s^−1^ using a Couette geometry (internal radius 16 mm, external radius 17 mm, cylinder length 33.35 mm), at 25 °C. At least three replicates were performed.

The extensional viscosity of the EKL/PVP solutions was measured at 25 °C using a CaBER 1 (Thermo Haake GmbH, Fellbach, Germany) extensional rheometer. A small drop of the solution was placed between two concentrically mounted plates (4 mm in diameter), which were then separated vertically, suddenly imposing tensile stress to generate an unstable flowing filament. The evolution of the filament diameter with time was monitored by means of a laser blade micrometer and balanced consideration of operating forces.

Surface tension measurements were carried out using a Sigma 703D (Biolin Scientific, Beijing, China) force tensiometer. The surface tension of each solution was measured with a platinum Wilhelmy plate (width 39.24 mm, thickness 0.1 mm) at 22 °C. Measurements were performed in triplicate.

The electrical conductivity values were determined with a CE GP31 high-frequency meter (Crison, Barcelona, Spain). The conductivity cell was previously calibrated with standard KCl solutions of known conductivity (1413 μS/cm and 12.88 μS/cm, respectively). Measurements were made with a universal platinum cell. At least three replicates were performed.

### 2.5. Characterization of Electrospun Nanostructures

Morphological characterization of EKL/PVP nanostructures was carried out by means of scanning electron microscopy (SEM) in a JEOL, model JXA-8200 SuperProbe, microscope operating at an acceleration voltage of 15 kV and different magnifications. Samples were previously gold-coated using a sputter coater HHV Scancoat Six SEM. The FIJI ImageJ analysis program was used to analyze the SEM images of the different electrospun nanostructures. For each image taken with the same magnification, 100 random image analyses were carried out.

### 2.6. Preparation and Rheological Characterization of Oleogels

The selected electrospun nanostructures were dispersed in castor oil, at 15 and 30 wt.% concentration (total weight sample: ~10–15 g) and room temperature, for 24 h, using an RW 20 IKA stirrer outfitted with an anchor-shaped agitator at 60 rpm. The resulting oleogels were stored at room temperature for further characterization.

Oleogels were rheologically characterized in a Rheoscope controlled-stress rheometer (Thermo Scientific, Waltham, MA, USA), using a serrate plate–plate geometry (20 mm diameter and 1 mm gap). Small-amplitude oscillatory shear (SAOS) tests were carried out in the linear viscoelastic regime, within a frequency range of 0.03–100 rad.s^−1^ at 25 °C. Stress sweep experiments were previously performed to determine the extension of the linear viscoelastic region. Viscous flow tests were also performed by applying stepped shear rate ramps (3 min in each step) within a shear rate range of 10^−2^–10^2^ s^−1^.

### 2.7. Statistical Analysis

An analysis of the variance (ANOVA) was performed using three replicates of each measurement independently. Then, a series of statistical parameters were calculated, including the mean and the standard deviation. Furthermore, a comparison of means tests was performed to detect significant differences (*p* < 0.05).

## 3. Results

### 3.1. Physico-Chemical Properties of EKL/PVP Solutions

**Figure 1:**
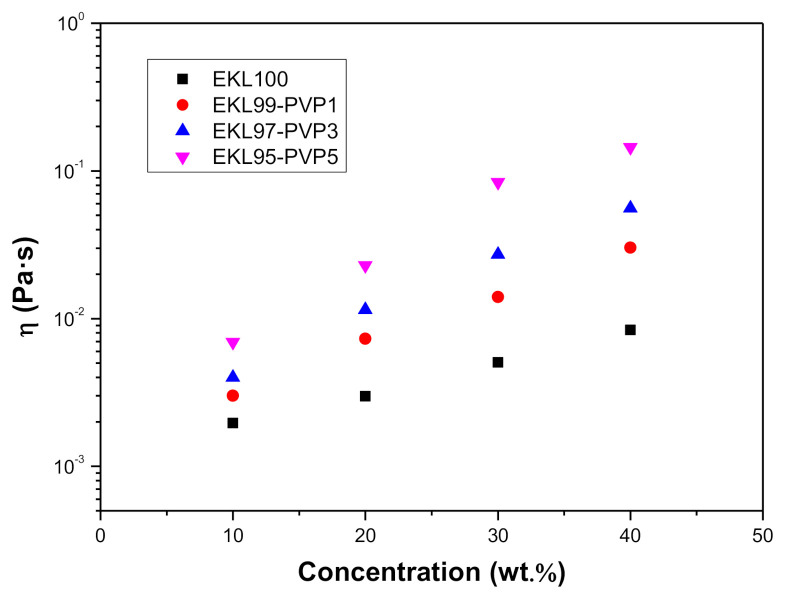
Shear viscosity of EKL/PVP solutions in DMF as a function of total polymer concentration and EKL:PVP ratio.

As reported elsewhere [24,26,45,46], the performance of the electrospinning process depends on several key physico-chemical properties of the electrospun solution such as viscosity, surface tension, and electrical conductivity. In particular, the solution viscosity has often been tailored by modifying the polymer concentration [47,48] and, indeed, some correlations were applied to find the minimum polymer concentration to achieve adequate viscosity values to perform the electrospinning process. For instance, Aslanzadeh et al. [49] determined that a certain threshold viscosity corresponding to the polymer overlap critical concentration is required to obtain relatively uniform nanofibers. Figure 1 shows the shear viscosity values for the different EKL:PVP solutions plotted vs. EKL/PVP concentration. All the EKL:PVP solutions studied exhibited a Newtonian behavior over the shear rate range applied (1–300 s^−^^1^). As can be observed, viscosity increases with EKL/PVP concentration and decreased with EKL:PVP ratio. As expected, the higher PVP proportion, the higher the viscosity value.

Apart from shear viscosity values, Table 2 presents the surface tension, electrical conductivity and extensional viscosity values of the electrospun solutions as a function of total polymer concentration and EKL:PVP ratio. As can be observed, a certain variability of the surface tension values is observed when modifying the EKL:PVP ratio, although lignin concentration exerts a more influential effect. Thus, an almost generalized increase in the surface tension values is observed as the solution concentration increases or the EKL:PVP ratio decreases. The surface tension of DMF was found to be 27.9 mN/m and the addition of EKL increased the surface tension, which could be attributed to improved interaction between DMF and kraft lignin. Teng et al. [33] reported that collapsed rigid spheres of lignin particles, rather than free isolated lignin segments, would be generated in DMF solutions due to intra- and intermolecular interactions between aromatic rings of lignin in DMF solutions containing lignin. This occurs since the attractive forces between lignin molecules are stronger than the dispersive forces of DMF molecules. DMF’s surface tension was, therefore, increased at higher lignin concentrations [50].

On the one hand, the analysis of the electrical conductivity data shows that EKL concentration has a significant influence on this physical property. Thus, at low EKL concentrations, a moderate increase in the polymer concentration, from 10 to 20 wt.% leads to a slight increase in the electrical conductivity of the solutions. This is could be due to the polar character of lignin whose complex chemical structure is composed of phenolic and aliphatic hydroxyl and carboxyl moieties and β-O-4′ alkyl–aryl ether-based substructures [51]. This slight increase in conductivity with EKL concentration indicates that these solutions are still below the overlap concentration, i.e., in the semi-diluted unentangled regime. However, the electrical conductivity decreased above those concentrations, presumably as a consequence of reduced mobility of the macromolecules [52,53], which suggests that the entanglement concentration of these solutions, i.e., the transition from the semi-diluted unentangled regime to semi-diluted entangled regime, occurs at around 20 wt.% EKL/PVP concentration. On the other hand, no clear influence of the EKL:PVP ratio on the electrical conductivity was observed.

In order to assess the kind of dilution regimes in these solutions and give more information about polymer–solvent interactions, a hydrodynamic study was performed. The intrinsic viscosity, which provides information on the ability of macromolecules to increase the viscosity of a solution in the absence of intermolecular interactions [54] was estimated and analyzed as a function of concentration, according to the following well-known relationships [55,56]:

(1) $$\eta_{rel}=\frac{\eta_{ps}}{\eta_{sol}}$$

(2) $$\eta_{sp}=\frac{\eta_{ps}-\eta_{sol}\;}{\eta_{sol}}=\eta_{rel}-1_{\;}$$

(3) $$\eta_{red}=\frac{\eta_{sp}}{C}\;\;\;{\;}_{\;}$$

(4) $$\left[\eta \right]=\underset{c\to 0}{\lim}\frac{\eta_{sp}}{c}$$

where η_rel_, η_sp_ and η_red_ are the relative, specific and reduced viscosities, respectively, while η_sol_ and η_ps_ refer to the viscosities of the solvent (DMF in this case) and the EKL/PVP solutions, respectively. Finally, [η] is the intrinsic viscosity. The most widely used methods to estimate the intrinsic viscosity are based on the Kraemer and Huggins models, given by Equations (5) and (6), respectively [57].

(5) $$\frac{\ln\eta_{rel}}{c}=\left[\eta \right]+k_{1}{\left[\eta \right]}^{2\;}c$$

(6) $$\eta_{red}=\;\left[\eta \right]+k_{2}\;{\left[\eta \right]}^{2}\;c$$

**Figure 2:**
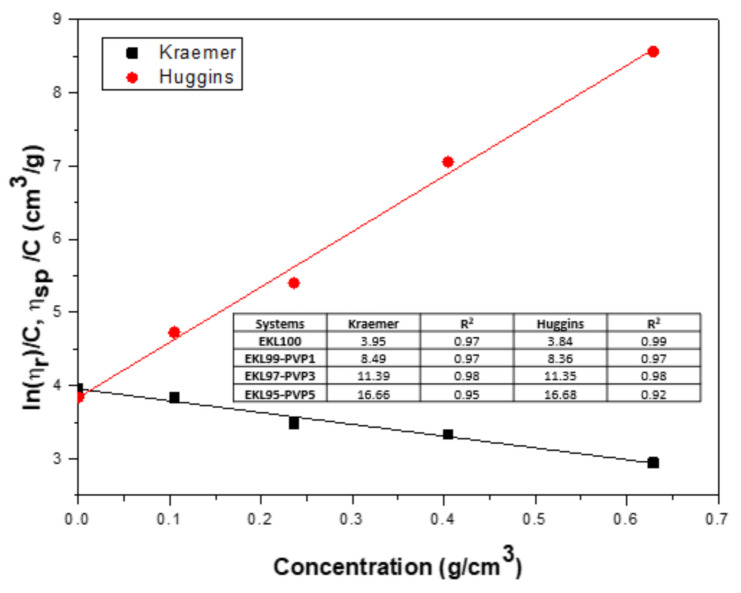
The Kraemer and Huggins plots for EKL/PVP solutions at a selected EKL:PVP ratio (100:0). The inset displayed the values of the intrinsic viscosity values for all the EKL:PVP ratios studied.

In this way, the relative and reduced viscosities for the different EKL/PVP solutions were calculated according to Equations (1) and (3) and plotted in the forms expressed in Equations (5) and (6), as illustrated in Figure 2 for concentrations comprised between 3 and 40 wt.%. Then, the intrinsic viscosity was estimated as the Y-intercept, i.e., when the concentration tends to zero [58]. As can be observed, both models adequately fitted the experimental data, resulting in values of intrinsic viscosity ranging from 4 to 17 cm^3^/g, depending on the EKL:PVP ratio (see the inset in Figure 2). It is well-known that the intrinsic viscosity provides information about the average interactions of single polymer molecules with the solvent and the polymer-specific hydrodynamic volume or average molecular weight [59]. These low values of the intrinsic viscosity suggest compact structures of EKL in DMF and relatively poor interactions with the solvent, according to the values of the Huggins constant, comprised between 0.50 and 0.66. Thus, the Huggins constant can be considered a measure of the solvent quality, indicating that the compatibility between lignin and DMF is not excellent, but is acceptable for lignins [60]. On the other hand, as expected, the intrinsic viscosity directly related to the average molecular weight increased with the addition of PVP.

**Figure 3:**
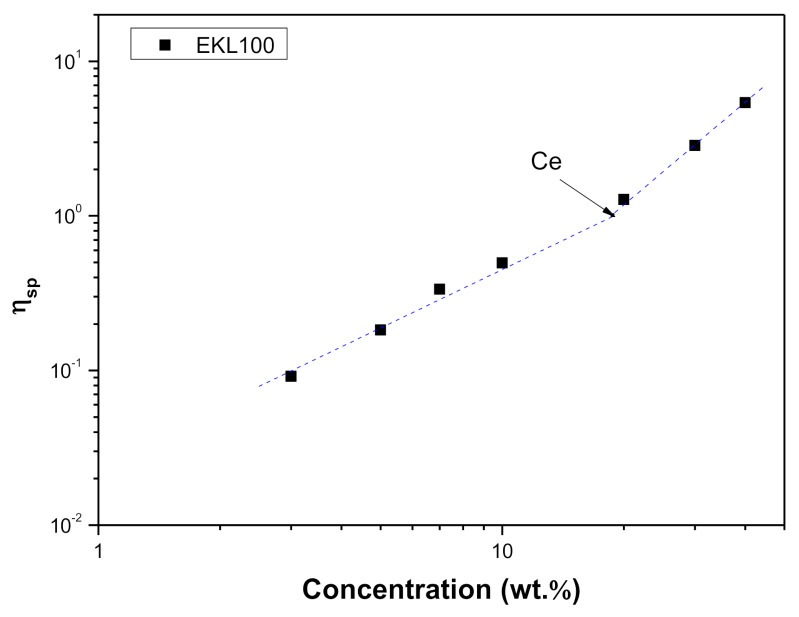
Specific viscosity vs. EKL concentration plot and estimation of the critical entanglement concentration.

Moreover, Figure 3 shows the relationship between the specific viscosity (η_sp_) and EKL concentration. The critical entanglement concentration (C_e_) delimiting the semi-diluted unentangled and the semi-diluted entangled regimes can be obtained as the change in the slope of this plot. As can be seen, C_e_ is 19.8 wt.%, from which an increase in the scaling exponent arose, from 1.3 to 2.1, which are values fairly in agreement with the expected values for a neutral polymer in the good solvent [61,62]. These experimental results corroborate what was previously hypothesized when discussing the effect of concentration on the electrical conductivity, which decreased above 20% concentration due to the reduced mobility of the macromolecules in the semi-dilute entangled regime.

**Figure 4:**
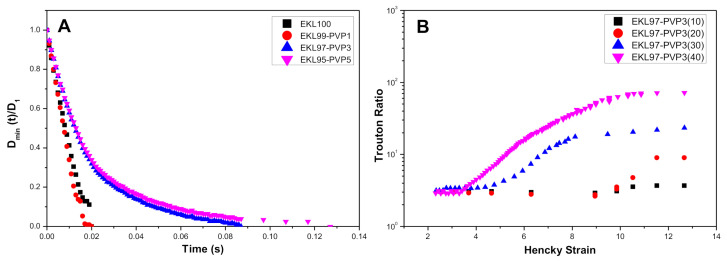
(**A**) Evolution of the normalized filament diameter with time, and (**B**) Trouton ratio versus Hencky strain plot obtained from the transient extensional experiments of EKL/PVP solutions, as a function of EKL:PVP ratio and EKL/PVP concentration, respectively.

The extensional viscosity of EKL:PVP solutions was also evaluated by means of capillary breakup experiments. Figure 4A illustrates the filament thinning profiles for different EKL/PVP solutions, where t = 0 corresponds to the time at which the upper plate stopped moving, and the midpoint filament diameter was then recorded by the laser micrometer as a function of time, D_min_ (t). Figure 4A shows the evolution of the D_min_(t) normalized by the initial filament diameter, D_0_, for solutions containing 40 wt.% of polymers at different EKL:PVP ratios. For EKL/PVP concentrations below 30 wt.%, the determination of the D_min_(t) is quite complicated due to sudden filament breakage or even the no filament formation, although it was possible in some cases, especially for the lowest EKL/PVP ratios, i.e., 3 and 5 wt.% PVP content. Dallmeyer et al. (2014) [63] reported similar behavior in solutions containing exclusively lignin at concentrations lower than 35 wt.%, showing that there is a limiting viscosity below that the filament formation is very committed. In this case, EKL:PVP solutions prepared with 30 and 40 wt.% showed an almost linear filament thinning evolution at high EKL/PVP ratios, i.e., 100:0 and 99:1, inherent to pure Newtonian fluids, whereas an exponential decay of the filament diameter, typical of viscoelastic liquids, was observed for lower EKL/PVP ratios, i.e., 97:3 and 95:5.

The extensional viscosity can be estimated from the filament thinning evolution by applying the following equation:

(7) $$\eta_{ext}=\frac{\sigma }{\frac{-d\;\left(D_{min}\left(t\right)\right)}{dt}}$$

where σ is the surface tension.

The extensional viscosity of these solutions remained reasonably constant with time during the first instants of the experiments, and then rose as a consequence of the increasing extensional deformation. The time (or strain) from which the viscosity began to increase was shortened by increasing either EKL/PVP concentration or the PVP content, as a result of an enhanced elastic character. The values of short time-limiting extensional viscosity (η_ext,0_) were provided in Table 2. Similar to that found with the shear viscosity, the extensional viscosity increased with EKL/PVP concentration and decreased with the EKL/PVP ratio. Figure 4B illustrates the effect of EKL/PVP concentration on the extensional viscosity expressed in the form of the Trouton ratio (η_ext_/η) vs. the Hencky strain (ε), estimated as follows:

(8) $$\varepsilon =-2\ln\left(\frac{D_{min}\left(t\right)}{D_{0}}\right)$$

For pure Newtonian fluids, the Trouton ratio should theoretically be equal to 3 and significantly higher for viscoelastic liquids [64,65]. As can be seen in Figure 4B, this theoretical value was accomplished in all cases at short times, and then Trouton ratio significantly increases over time, especially for the more concentrated solutions and for the lower EKL:PVP ratios, reaching values up to 80–90. These results point out the convenience of performing both extensional and shear flow tests to assess the electrospinnability of biopolymer solutions, since shear experiments provided only Newtonian responses, whereas extensional tests revealed the viscoelastic character, which may be of decisive importance in the electrospinning process [66].

### 3.2. Characterization of EKL/PVP Electrospun Nanostructures

**Figure 5:**
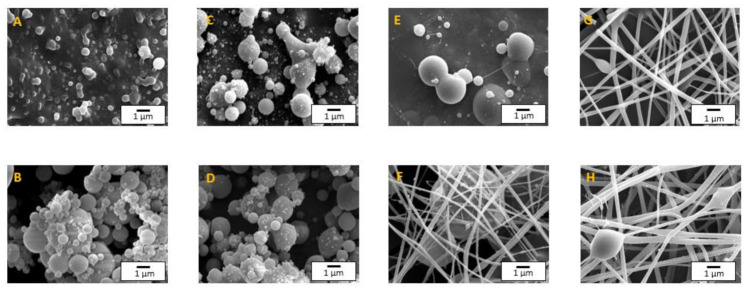
SEM electrospun nanostructures obtained at different EKL:PVP ratios. (**A**) EKL100 at 30 wt.%., (**B**) EKL100 at 40 wt.%., (**C**) EKL99-PVP1 at 30 wt.%., (**D**) EKL99-PVP1 at 40 wt.%., (**E**) EKL97-PVP3 at 30 wt.%., (**F**) EKL97-PVP3 at 40 wt.%., (**G**) EKL95-PVP5 at 30 wt.% and (**H**) EKL95-PVP5 at 40 wt.%.

Figure 5 shows the micrographs of the different electrospun nanostructures obtained by SEM from solutions at 30 and 40 wt.% and several EKL:PVP ratios. As can be noticed, the electrospinning of PVP-free solutions was not able to generate lignin interconnected nanofibers, but instead, the resulting membranes consisted of agglomerated micro-sized particles (Figure 5A,B). Something similar occurred when only a small amount of PVP (99:1 EKL:PVP ratio) was added to dope the lignin solutions, as can be seen in Figure 5C,D.

When decreasing the EKL:PVP ratio to 97:3, morphologies dealing with micro-and nano-sized particles either connected by few thin filaments or embedded in nanofiber mats were predominantly obtained from solutions at 30 wt.% and 40 wt.% EKL/PVP, respectively (Figure 5E,F). Finally, uniform nanofiber mats with a few beaded fibers randomly distributed were obtained by raising PVP proportion, i.e., 95:5 EKL:PVP ratio (Figure 5G,H). These results are in agreement with those obtained in other studies [24,66,67], in which the addition of a dopant polymer was reported to be essential for the generation of bead-free and/or uniform kraft lignin nanofibers by electrospinning. It is noteworthy that nanofibers were not generated from solutions containing 10 and 20 wt.% EKL/PVP total concentration regardless of the EKL:PVP ratio, but only electrosprayed particles. In this sense, the formation of nanofibres seems to be conditioned to exceeding C_e_ in the feed solution.

Table 3 shows the particle and fiber mean diameters of the nanostructures obtained by electrohydrodynamic processing. As can be observed, there is an increase in the mean size of the particles and fibers by increasing the total polymer concentration or, especially, by decreasing the EKL:PVP ratio. Some authors observed a similar tendency with the fiber mean diameter for electrospun lignin nanofiber mats, using different types of lignins and dopant polymers, such as PEO, PVA, or PAN [24,63,67,68,69]. As mentioned above, there is a relationship between the concentration of the polymer and the viscosity of the solution used in the electrospinning process. Dallmeyer et al. (2014) [66] found a relationship between the terminal relaxation time and the mean fiber diameter, concluding that the rheological properties of the spinning solution influence the mean fiber diameter as well as the size distribution of the fibers obtained. In this study, relatively uniform nanofiber mats were only obtained from solutions having η_ext,0_ values of at least 0.20 Pa s.

**Figure 6:**
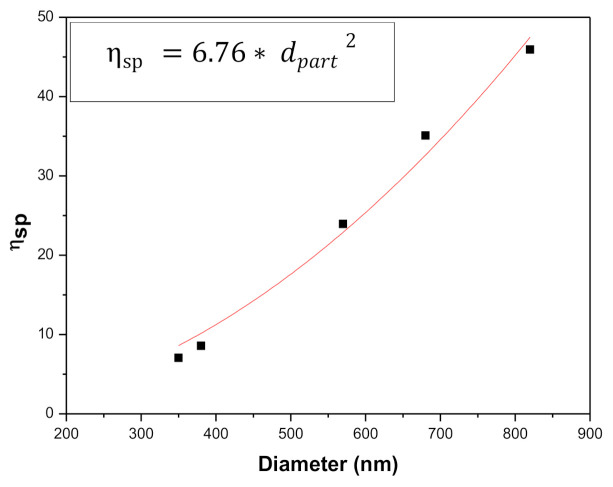
Correlation of the specific viscosity of EKL/PVP solutions and average diameter of resulting electrosprayed particles.

Moreover, as Figure 6 illustrates, the specific viscosity of the spinning solution correlates with the average size of the electrosprayed particles when the extensional properties of the solution are not high enough to generate nanofibers. For these systems, the required specific viscosity value of the solution to obtain a certain mean particle diameter can be predicted from the empirical correlation included in the inset of Figure 6.

### 3.3. Ability of EKL/PVP Nanofibers to form Oleogels

According to the morphological properties of the different structures generated, EKL nanofiber templates obtained from solutions at 30 and 40 wt.% using PVP as dopant polymer, at 95:5 EKL:PVP ratio, were selected to assess the ability to form physically stable oleogels upon dispersion in castor oil. In principle, these nanostructures exhibiting high porosity and high surface area/volume ratio may be suitable for entrapping the vegetable oil and thus stabilizing the resulting gel-like system. These EKL/PVP nanostructures were simply dispersed in castor oil under gentle agitation at 15 and 30 wt.% yielding stable oleogels. It is worth mentioning that dispersing other kinds of structures mainly comprised of micro-sized particles results in phase separation almost immediately. This fact confirms that the generation of three-dimensional networks with large specific surface areas is essential for enhancing the physical interactions between the oil and the nanofibers.

**Figure 7:**
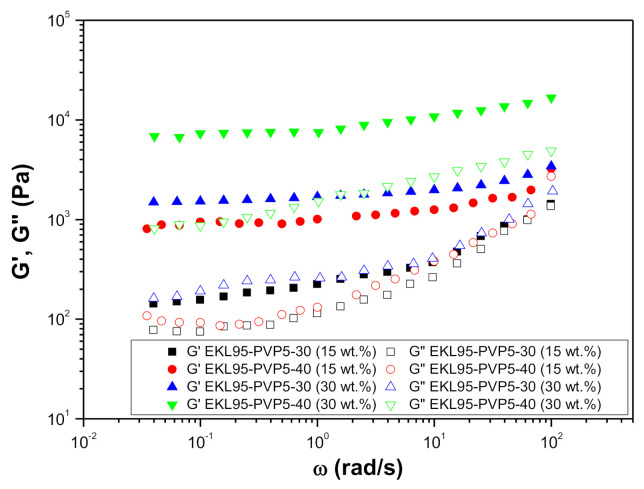
Evolution of the linear viscoelastic functions with frequency for oleogels prepared with EKL/PVP electrospun nanofibers (applied stress: 1–3 Pa, depending on the consistency of the sample).

Figure 7 shows the mechanical spectra of the resulting oleogels as a function of EKL/PVP concentration in the original spinning solution and nanofiber concentration in the oil medium. The evolution of the SAOS functions with frequency is qualitatively similar for all the oleogels, as the storage modulus, G′, is always higher than the loss modulus, G”, in the whole frequency range studied. In addition, a plateau region can be noticed at low and intermediate frequencies, whereas a tendency to reach a crossover point between both moduli may be generally observed at high frequencies. This is particularly relevant for the system with 15 wt.% nanofiber concentration obtained from spinning solutions at 30 wt.% EKL/PVP, which exhibited very similar values of the storage and loss moduli at medium and high frequencies, thereby reflecting a low relative elasticity. Moreover, the values of SAOS functions increased using electrospun nanofibers obtained from more concentrated solutions, i.e., 40 wt.% vs. 30 wt.% EKL/PVP, and especially when increasing the amount of nanofibers added to the oil medium. In this sense, bearing in mind a potential application as bio-based lubricating grease formulations, the rheological properties, i.e., the consistency, of these oleogels can be tailored depending on nanofiber concentration, electrospinning processing conditions and nanofiber morphology. For instance, it is well-known that typical NLGI grade 1–2 (high-medium consistency grades) lubricating greases usually present G′ values of around 10^4^ Pa and G” values around one order of magnitude lower [70,71], as shown by the most concentrated oleogel prepared with EKL95-PVP5-40 nanofibers, whereas softer greases generally display lower values of the SAOS functions.

**Figure 8:**
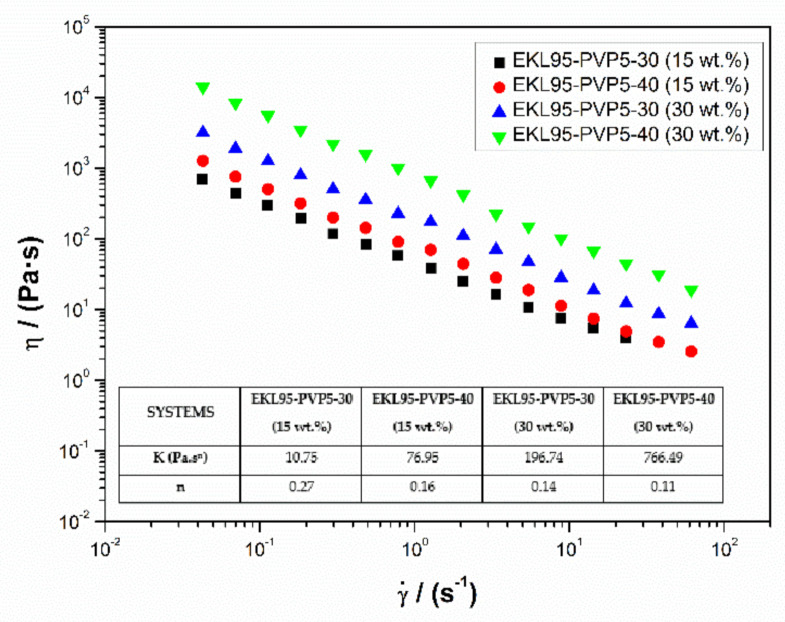
Viscous flow curves of oleogels prepared with electrospun EKL/PVP nanofibers.

Figure 8 shows the viscous flow curves of the EKL/PVP nanofiber-stabilized oleogels. In all cases, the power-law model fits fairly well the shear thinning behaviour observed [51]:

(9) $$\eta =k\;{\dot{\gamma }}^{n-1}$$

where *k* is the consistency index and *n* is the flow index. *k* and *n* values are listed in the inset of Figure 8 for the different oleogels studied. As can be observed, the viscosity was influenced by nanofiber concentration and by the concentration of the spinning solution in a similar way than previously discussed for the SAOS functions. Thus, *k* increased with the percentage of nanofibers incorporated into the oleogel and by the percentage of EKL/PVP in the spinning solution. On the other hand, the values of the flow index, *n*, are between 0.27 and 0.11, resulting in markedly shear-thinning characteristics, as traditionally found in lithium lubricating greases [71,72,73,74]. In general, depite nanofiber concentration exerting a major influence on the rheological properties of the oleogel, these properties can also be modulated considering the inter-related and/or cumulative effect of the polymer concentration in the spinning solution. For instance, the red and blue curves in Figure 7 and Figure 8 are not so different for oleogels containing different nanofiber concentrations.

## 4. Conclusions

Different micro- and nano-structures were prepared by means of electrohydrodynamic processing from solutions of eucalyptus kraft lignin (EKL), doped to some extent with polyvinylpyrrolidone (PVP), in DMF. Solutions containing 10 and 20 wt.% EKL/PVP total concentration were not able to produce nanofibers but only electrosprayed particles, whereas at higher concentrations (30 and 40 wt.%) morphologies dealing with either micro- and nano-sized particles embedded in nanofiber mats or uniform nanofiber mats with a few beaded fibers were obtained, especially at EKL:PVP ratios lower than 97:3, as a consequence of the increased extensional properties and reaching the semi-dilute entangled regime. A hydrodynamic study was performed and the intrinsic viscosity was estimated from the fitting of experimental data to the Kraemer and Huggins models. Intrinsic viscosity values ranged from 4 to 17 cm^3^/g, depending on the EKL:PVP ratio. The morphology of the electrospun nanostructures was strongly dependent on both the proportion of EKL/PVP employed and the solution concentration. The specific viscosity of the spinning solution correlates with the average size of the electro-sprayed particles when the extensional properties of the solution are not high enough to generate nanofibers. Different physically stable oleogels were unprecedently developed by simply dispersing EKL/PVP electrospun nanofibers in castor oil, at nanofiber concentrations above 15 wt.%, without needing any other physical or chemical pretreatment. On the contrary, micro-sized particles did not generate stable oleogels. Nanofiber templates with high porosity and a high surface-to-volume ratio are suitable for the entrapment of the vegetable oil and hence induce stabilization of the resulting gel-like systems. The rheological properties of oleogels can be tailored by modifying the concentration of nanofibers and/or the polymer concentration in the spinning solution. Both variables exerted a combined and inter-related influence on the rheological properties of these oleogels. Overall, the electrospinning of EKL/PVP solutions can be proposed as a method to produce nanostructures which enable oil thickening, leading to oleogels with potential applications in the lubricant industry. The application of lignin as a thickening agent in the manufacture of lubricating greases is justified as a new way of valorizing of lignocellulosic waste, combining ecological, economic and social benefits. Finally, a more in-depth rheological characterization and tribological studies will be required in the future to further validate these oleogels as effective substitutes for traditional lubricating greases.

## Figures and Tables

**Table 1:** Nomenclature and concentration for samples studied.

Sample Code	EKL (wt.%)	PVP (wt.%)
EKL100	100	0
EKL99-PVP1	99	1
EKL97-PVP3	97	3
EKL95-PVP5	95	5

**Table 2:** Surface tension, electrical conductivity and shear and extensional viscosity for EKL:PVP solutions in DMF. Values with different symbols are significantly different (*p* < 0.05).

Concentration (wt.%)	EKL:PVP Ratio	Surface Tension (mN/m)	Electrical Conductivity (μS/cm)	η (mPa.s)	η_ext,0_ (mPa.s)
10	100:0	29.55 ^a^	190.1 ^A^	2.9 ^α^	7.1 ^aa^
	99:1	30.01 ^a^	181.5 ^B^	3.3 ^α^	10.2 ^bb^
	97:3	31.05 ^b^	215.9 ^C^	4.2 ^β^	15.3 ^cc^
	95:5	31.25 ^b^	189.4 ^A^	7.7 ^γ^	23.4 ^dd^
20	100:0	31.34 ^b^	201.1 ^D^	3.4 ^α^	9.8 ^bb^
	99:1	32.35 ^c^	201.7 ^D^	8.1 ^γ^	24.1 ^dd^
	97:3	32.73 ^c^	210.8 ^C^	12.5 ^δ^	41.9 ^ee^
	95:5	33.33 ^d^	176.3 ^B^	23.1 ^ε^	88.7 ^ff^
30	100:0	31.01 ^a^	166.6 ^E^	5.1 ^β^	15.4 ^cc^
	99:1	33.48 ^d^	167.4 ^E^	14.2 ^δ^	45.8 ^gg^
	97:3	34.76 ^e^	150.2 ^F^	27.3 ^ζ^	90.5 ^ff^
	95:5	34.95 ^e^	170.1 ^E^	84.7 ^ᵨ^	299.7 ^hh^
40	100:0	34.75 ^e^	96.3 ^G^	8.2 ^γ^	32.7 ^ii^
	99:1	36.27 ^f^	111.5 ^H^	30.2 ^ζ^	118.8 ^jj^
	97:3	38.05 ^g^	95.2 ^G^	56.4 ^ᵩ^	205.1 ^kk^
	95:5	35.45 ^e^	101.8 ^G^	145.5 ^ᵪ^	445.8 ^ll^

Different symbols, included as superscripts (a, b, c, d, e, f, g; A, B, C, D, E, F, G, H; α, β, γ, δ, ε, ζ, ᵩ, ᵪ; aa, bb, cc, dd, ee, ff, gg, hh, ii, jj, kk, ll), indicate significant differences (*p* < 0.05) in each column.

**Table 3:** Average particle and fiber diameters of the different micro- and nano-structures obtained by electrohydrodynamic processing.

Concentration (wt.%)	Samples	Particle Diameter (nm)	Fiber Diameter (nm)
30	EKL100	350	-
	EKL99-PVP1	570	-
	EKL97-PVP3	820	125
	EKL95-PVP5	-	710
40	EKL100	380	-
	EKL99-PVP1	680	-
	EKL97-PVP3	-	585
	EKL95-PVP5	-	820

## Data Availability

Not applicable.
